# Prognosis Associated with Glycolytic Activity in Regional Lymph Nodes of Patients with Previously Untreated Metastatic Breast Cancer: A Preliminary Study

**DOI:** 10.3390/diagnostics12081809

**Published:** 2022-07-27

**Authors:** Hojin Cho, Arthur Cho, Won Jun Kang

**Affiliations:** Department of Nuclear Medicine, Severance Hospital, Yonsei University College of Medicine, Seoul 03722, Korea; hojincho@yuhs.ac (H.C.); artycho@yuhs.ac (A.C.)

**Keywords:** breast cancer, prognosis, lymph node, fluorodeoxyglucose, positron emission tomography

## Abstract

Better mechanisms of predicting prognoses in patients with metastatic breast cancer will improve the identification of patients for whom curative treatments may be the most effective. In this study, the prognostic value of ^18^F-fluorodeoxyglucose positron emission tomography/computed tomography (^18^F-FDG PET/CT) was assessed in patients with metastatic breast cancer. A retrospective analysis of women who underwent ^18^F-FDG PET/CT for staging of newly diagnosed metastatic breast cancer was conducted. In each patient, the maximum standardized uptake value (SUV) and total lesion glycolysis (TLG) of primary tumors and regional lymph nodes were measured and analyzed for association with survival using the Cox proportional hazards regression model. From 346 consecutive patients, 32 with metastatic invasive ductal carcinoma of the breast were included in the study. The median duration of follow-up was 22.5 months. Disease progression occurred in 26 patients, and 11 patients died. When multivariate analyses with a stepwise forward regression were applied, only the maximum SUV and TLG of regional lymph nodes showed a significant correlation with progression-free survival and overall survival, respectively. This study demonstrates that increased ^18^F-FDG uptake in regional lymph nodes is a strong independent predictor of survival in women with metastatic invasive ductal carcinoma of the breast.

## 1. Introduction

Approximately 6–10% of patients with breast cancer present with metastatic disease at the time of the diagnosis, and approximately 30% of patients initially diagnosed with earlier stages of breast cancer will eventually develop recurrent advanced or metastatic disease [1]. Despite therapeutic advances, only modest survival improvements have been observed. For women with metastatic breast cancer at diagnosis, median survival is reported to be 2–4 years [2,3,4,5], although in small populations long-term survival has been observed [6]. Current prognostic markers are limited in their ability to predict clinical outcomes with accuracy. Enhancing the formulation of prognoses in patients with metastatic breast cancer will improve the selection of the patients for whom curative treatments can be the most effective.

An analog of glucose, ^18^F-fluorodeoxyglucose (^18^F-FDG) can aid in the visualization of elevated glycolysis commonly seen in tumors. ^18^F-FDG positron emission tomography/computed tomography (PET/CT) has been evaluated for detection, diagnosis, staging locoregional and distant sites, monitoring responses to chemotherapy, and prognostication in patients with breast cancer [7]. Current guidelines recommend conventional imaging including bone scintigraphy, ultrasound, CT, and magnetic resonance imaging (MRI) for staging evaluation of patients with metastatic breast cancer [8,9]. However, it is not clear whether ^18^F-FDG PET/CT will provide prognostic information in this setting. Only few studies have assessed the prognostic role of ^18^F-FDG PET/CT in patients with metastatic breast cancer, and the prognostic significance of baseline ^18^F-FDG PET/CT was inconclusive [10,11]. In this study, we retrospectively assessed the prognostic value of baseline ^18^F-FDG PET/CT in patients with metastatic breast cancer.

## 2. Materials and Methods

### 2.1. Patients

From June 2005 through December 2008, we retrospectively reviewed the medical records of all female patients who underwent ^18^F-FDG PET/CT for staging of breast cancer at our institution. Patients were eligible if they had newly diagnosed, histologically proven breast cancer, had not received any therapy, and had distant metastasis. A patient was considered positive for lymph node or distant metastasis under the following conditions: histological or cytological evidence; typical features of metastasis on at least two different imaging modalities; or an equivocal lesion with apparent progression on follow-up studies. Patients were excluded if they had other malignancies or bilateral breast cancer. Disease status was assessed according to the Response Evaluation Criteria in Solid Tumors 1.1 [12] at baseline and every 9 to 12 weeks until progression. The requirement for informed consent for this study was waived by the institutional review board.

**Figure 1:**
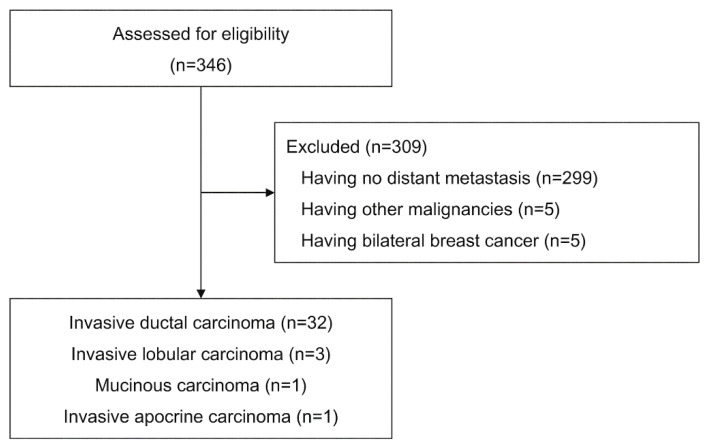
Flow diagram for patient inclusion.

During the study period, 346 consecutive potentially eligible patients were identified. Patients were excluded for the following reasons: no distant metastasis (299 patients), other malignancies (5 patients), and bilateral breast cancer (5 patients). Of the remaining 37 patients, 32 had invasive ductal carcinoma, three had invasive lobular carcinoma, one had mucinous carcinoma, and one had invasive apocrine carcinoma. Patients who had histologic types other than invasive ductal carcinoma were excluded from further analysis due to their small sample sizes (Figure 1).

The median age of the remaining 32 patients was 52.5 years (range, 34 to 75 years), and Eastern Cooperative Oncology Group scores for performance status was 0 or 1 in 29 patients (90.6%). Visceral disease (lung, liver, brain, and other organs) was present in 21 patients (65.6%) and 23 patients (71.9%) had three or more metastatic sites. Three patients (9.4%) started hormonal therapy or immunotherapy alone and 29 patients (90.6%) received chemotherapy (alone or in combination with other treatments) as their first-line of therapy for metastatic disease (Table 1). The presence of metastatic disease was confirmed either by histological or cytological findings in six patients or by multiple imaging modalities with clinical and imaging follow-up in 26 patients. Patients were followed for a median of 22.5 months (range, 2–51 months). Progression of disease was observed in 26 patients (81.3%), and 11 patients (34.4%) died during the follow-up period.

### 2.2. PET/CT Imaging

All patients were instructed to fast for at least 6 h, and their plasma glucose concentrations were measured before the intravenous administration of 370–555 MBq of ^18^F-FDG. The blood glucose level was less than 7.8 mmol/L. One hour later, images were obtained on either a Gemini (Philips Medical Systems) or a DSTe (GE Healthcare) integrated PET/CT scanner. A low-dose CT scan was obtained for attenuation correction using the following parameters for the Gemini: 120 kVp, 50 mAs, 0.5-s rotation time, 5.0-mm scan reconstruction, 60-cm field of view, and a 512 × 512 matrix; and the following parameters for the DSTe: 140 kVp, 30 mA, 0.8-s rotation time, 3.3-mm scan reconstruction, 50-cm field of view, and a 512 × 512 matrix. A PET scan was then immediately acquired from the head to the mid-thigh level in three-dimensional mode at 3 min per bed position. A diagnostic CT scan was acquired using standard protocols (120 kVp, 250 mAs, 0.5-s rotation time, 3.0-mm scan reconstruction, 50-cm field of view, and a 512 × 512 matrix for the Gemini; 140 kVp, 230 mA, 0.8-s rotation time, 3.3-mm scan reconstruction, 50-cm field of view, and a 512 × 512 matrix for the DSTe; with 2 milliliter per kilogram of body weight of intravenous contrast medium containing 300 mg of iodine per milliliter (Omnipaque, Iohexol; GE HealthCare), administered at a rate of 1.5–2.0 milliliter per second), followed by the PET scan. The PET images were reconstructed using an iterative reconstruction algorithm based on the ordered-subset expectation maximization.

### 2.3. Image Analysis

**Figure 2:**
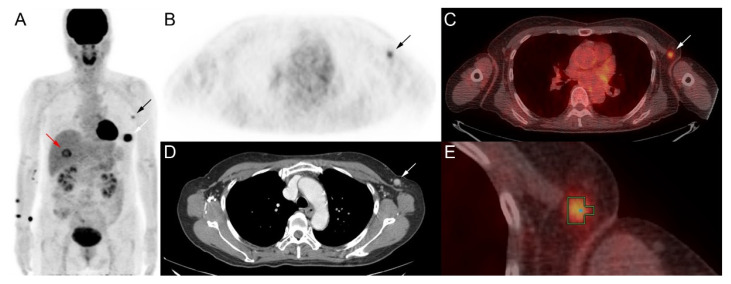
A 60-year-old woman with invasive ductal carcinoma of the left breast. The patient had biopsy-proven metastases of the left axillary lymph node and liver. Maximum intensity projection image of ^18^F-fluorodeoxyglucose positron emission tomography (^18^F-FDG PET) (**A**) shows abnormally increased ^18^F-FDG uptake in the left breast (white arrow), left axilla (black arrow) and liver (red arrow). Trans-axial PET image (**B**), fused positron emission tomography/computed tomography (PET/CT) image (**C**), and contrast-enhanced CT image (**D**) show an enlarged lymph node with increased ^18^F-FDG uptake in the left axilla (arrows). Volume of interest was drawn around the left axillary lymph node with a threshold of 50% maximum standardized uptake value on fused PET/CT image (**E**). The maximum standardized uptake value and total lesion glycolysis of the left axillary node were 2.51 and 1.16, respectively. The patient survived without progression during the follow-up period of 33 months.

PET/CT images were reviewed on a workstation (Advantage Workstation 4.4; GE Healthcare). Volumes of interest (VOI) were drawn around primary tumors and regional lymph nodes using an automatic iso-contour with a threshold of 50% maximum standardized uptake value (SUV) in the VOI (Figure 2), as previously described [13]. Contrast-enhanced CT images were used as an anatomical reference. If multiple lymph nodes were involved, the lymph node with the maximum ^18^F-FDG uptake was chosen. The total lesion glycolysis (TLG) was defined as a product of the average SUV and lesion volume. In each lesion, the maximum SUV and TLG were measured.

### 2.4. Statistical Analysis

Progression-free survival was defined as the time between diagnosis and either disease progression, or death due to any cause. Overall survival was defined as the time between diagnosis and death. If neither progression nor death occurred during the follow-up period, the patient was censored at the date of last follow-up visit.

Relative risks and 95% confidence intervals were calculated with univariate and multivariate Cox proportional-hazards regression models. The proportional-hazards assumption was assessed by using the Schoenfeld residuals [14]. Multivariate analyses were performed with a stepwise forward regression model and Akaike’s Information Criterion (AIC) [15], in which each variable with a *p* value of less than 0.05 (based on the univariate analysis) was entered into the model. The best-fit models were selected based on the smallest values of AIC. If continuous variables were found to be significant in the final model, they were dichotomized according to the cutoff value that yielded the smallest AIC value. Before dichotomization, continuous variables with skewed distribution were logarithmically transformed to fit a normal distribution. The log-rank test was used for comparisons of Kaplan-Meier curves. All statistical tests are two-sided. Statistical analyses were performed using SPSS software (version 18.0.2).

## 3. Results

### 3.1. Image Analysis

**Figure 3:**
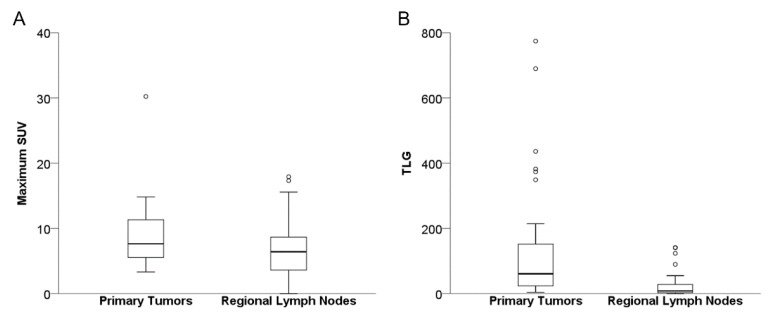
Box plot of ^18^F-FDG uptake of primary tumors and regional lymph nodes. Panels show the maximum standardized uptake value (SUV) (**A**) and total lesion glycolysis (TLG) (**B**) of primary tumors and regional lymph nodes. Circle represents outliers.

The median maximum SUV and TLG of primary tumors were 7.65 (range, 3.32–30.23) and 60.96 (range, 3.83–774.44), respectively. The median maximum SUV and TLG of regional lymph nodes were 6.43 (range, 0–17.93) and 8.56 (range, 0–141.65), respectively (Figure 3).

### 3.2. Analysis of Prognostic Factors

In the univariate analysis, only the maximum SUV and TLG of regional lymph nodes were significantly associated with both progression-free survival and overall survival. Clinical nodal stage and the maximum SUV of primary tumors were significantly associated with progression-free survival (Table 2). Thus, multivariate analysis for progression-free survival was performed with clinical nodal stage, the maximum SUV of primary tumors, and the maximum SUV and TLG of regional lymph nodes. Multivariate analysis for overall survival was assessed with the maximum SUV and TLG of regional lymph nodes.

**Figure 4:**
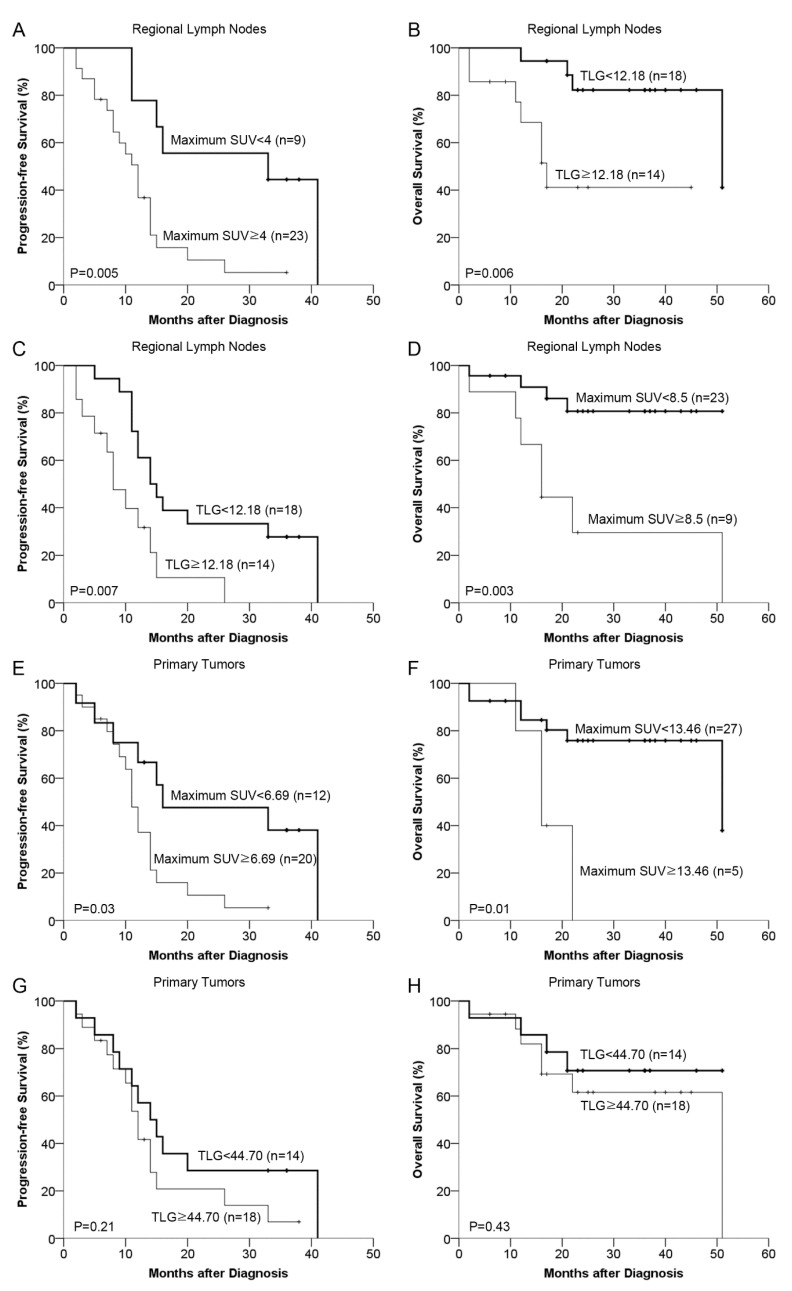
Kaplan-Meier estimates of survival according to ^18^F-FDG uptake. Based on multivariate cox regression analysis, progression-free survival (**A**) and overall survival (**B**) are shown for two subgroups dichotomized according to the maximum standardized uptake value (SUV) and total lesion glycolysis (TLG) of regional lymph nodes, respectively. The cutoff points for the maximum SUV and TLG of regional lymph nodes were 4.00 and 12.18 (the exponential of 2.5), respectively. Other possible combinations are also shown for comparison (**C**–**H**). The cutoff points were 12.18 (the exponential of 2.5), 8.5, 6.69 (the exponential of 1.9), 13.46 (the exponential of 2.6), 44.70 (the exponential of 3.8) and 44.70, respectively. Tick marks indicate times of censoring.

In the multivariate analysis, the only statistically significant independent prognostic factors for progression-free survival and overall survival were the maximum SUV (<4.00 vs. ≥4.00; hazard ratio, 3.77; 95% confidence interval [1.36, 10.48]; *p* = 0.01) and TLG (<12.18 vs. ≥12.18; hazard ratio, 5.64; 95% confidence interval [1.41, 22.52]; *p* = 0.01) of regional lymph nodes, respectively. The cutoff value for total lesion glycolysis of regional lymph nodes was determined after natural logarithmic transformation due to the skewed nature of the data. Based on the AIC, the best cutoff values of the maximum SUV and TLG of regional lymph nodes were 4.00 and 12.18, respectively. In Figure 4, Kaplan-Meier curves demonstrate progression-free survival and overall survival. Rates of progression-free survival and overall survival were significantly lower in patients with higher maximum SUV and TLG of the regional lymph nodes (*p* = 0.005 and *p* = 0.006, respectively).

## 4. Discussion

Our study demonstrated that a single ^18^F-FDG PET/CT performed at the time of the diagnosis in women with metastatic invasive ductal carcinoma of the breast is a strong prognostic indicator for survival. Uptake of ^18^F-FDG in the regional lymph nodes was a strong independent predictor of survival. The maximum SUV and TLG of the regional lymph nodes significantly correlated with progression-free survival and overall survival, respectively. In contrast, the maximum SUV and TLG of primary tumors did not show a significant correlation with progression-free survival or overall survival in the final model.

Patients with metastatic breast cancer comprise a heterogeneous group whose prognosis and outcome can vary depending on various factors, such as age, hormone-receptor status, and extent of the disease. Even in a single patient, intertumoral heterogeneity is common. Heterogeneity between the receptor status and genetic alterations of primary and metastatic breast cancer has been well described [16,17,18,19]. Conventional prognostic markers for metastatic breast cancers are largely based on anatomic staging and may have reached their limit in predicting outcomes [20,21]. Thus, ^18^F-FDG PET/CT may serve as a significant prognostic indicator that is needed for this group of patients.

Increased glycolysis has long been recognized as a hallmark of cancer [22]. The glycolytic phenotype has been shown to be associated with more aggressive tumor growth and increased metastatic potential in breast cancer [23,24]. Uptake of ^18^F-FDG by primary tumors has been demonstrated to correlate with prognostic indicators such as histologic type and indices of cellular proliferation. Prognostic factors such as tumor size, axillary node status, and steroid receptor status generally do not correlate with ^18^F-FDG uptake [25,26,27,28,29]. Correlative studies suggest that ^18^F-FDG PET/CT provides information on biological behavior of breast cancer that is independent of established prognostic factors.

As a heterogeneous disease, high intra- and intertumoral variability of ^18^F-FDG uptake has been observed in breast cancer [27,30,31]. Previous studies have evaluated the association between ^18^F-FDG uptake and prognosis. Patients with higher ^18^F-FDG uptake by the primary tumor have been shown to have poor outcomes [25]. A few studies also have addressed the uptake of ^18^F-FDG by the regional lymph nodes and their association with prognosis in patients with nonmetastatic breast cancer [32,33,34]. However, the parameters derived from ^18^F-FDG uptake such as maximum SUV, metabolic tumor volume, and total lesion glycolysis were inconsistent in predicting prognosis, and may reflect various aspects of tumoral heterogeneity [35,36,37].

Breast cancer metastasis occurs predominantly via the lymphatic system, and the extent of lymph node involvement is a pivotal prognostic indicator for the disease [38,39]. The presence of metastasis to regional lymph nodes on histologic examinations provides proof that primary tumors have acquired the ability to metastasize, and may have metastasized to distant sites as well. Although the presence of axillary node metastases in breast cancer is the most important prognostic indicator in operable cases, the fact that 20–30% of patients without axillary metastasis are not cured by local-regional therapy and some patients with metastasis have prolonged survival suggests that nodal metastases are substandard indicator in systemic disease [40,41,42]. The fact that ^18^F-FDG PET/CT provides noninvasive and highly reproducible quantitative means of measuring glycolysis in a desired region [13] points to its value for stratifying the clinical significance of regional lymph node metastases.

The limitations of this study are mainly due to the retrospective nature of the patient selection, which may lend itself to a possible selection bias, and the lack of a validation cohort. This study is a single-center study, which may affect reproducibility. Additionally, because the numbers of patients were small, subgroup analysis was not possible.

## 5. Conclusions

This study demonstrates that increased ^18^F-FDG uptake of regional lymph nodes is a strong independent predictor of survival in women with metastatic invasive ductal carcinoma of the breast. Larger prospective studies are needed to validate the significance of increased glycolysis of regional lymph nodes in this population of patients.

## Figures and Tables

**Table 1:** Demographic and clinical characteristics of the patients.

Characteristic	All Patients (*n* = 32)
Age at diagnosis—years	
Median	52.5
Range	34–75
Menopausal status—no. (%)	
Premenopausal	15 (46.9)
Postmenopausal	17 (53.1)
ECOG performance status—no. (%)	
0	16 (50.0)
1	13 (40.6)
2	3 (9.4)
Estrogen-receptor and progesterone-receptor status—no. (%)	
Positive for either	23 (71.9)
Negative for both	9 (28.1)
HER2 status—no. (%)	
Positive	12 (37.5)
Negative	20 (62.5)
Tumor stage—no. (%)	
T1	4 (12.5)
T2	15 (46.9)
T3	4 (12.5)
T4	9 (28.1)
Nodal stage—no. (%)	
N0	1 (3.1)
N1	15 (46.9)
N2	0 (0.0)
N3	16 (50.0)
Extent of disease—no. (%)	
<3 sites	9 (28.1)
≥3 sites	23 (71.9)
Sites of metastasis—no. (%)	
Visceral	21 (65.6)
Non-visceral only	11 (34.4)
Type of therapy—no. (%)	
Hormone therapy, immunotherapy, or both	3 (9.4)
Chemotherapy alone or combined with other therapy	29 (90.6)

ECOG, Eastern Cooperative Oncology Group; HER2, human epidermal growth factor receptor type 2.

**Table 2:** Univariate Cox regression models of progression-free survival and overall survival.

Variable	Progression-Free Survival		Overall Survival	
	Hazard Ratio (95% CI)	*p* Value	Hazard Ratio (95% CI)	*p* Value
Age at diagnosis—years *	1.02 (0.98–1.06)	0.34	1.03 (0.96–1.09)	0.45
Menopausal status				
Premenopausal	0.80 (0.36–1.77)	0.58	0.78 (0.24–2.57)	0.69
Postmenopausal	1.00		1.00	
ECOG performance status				
0	1.00		1.00	
1	1.89 (0.80–4.44)	0.15	1.00 (0.27–3.73)	1.00
2	3.41 (0.91–12.78)	0.07	4.54 (0.85–24.16)	0.08
Estrogen-receptor and progesterone-receptor status				
Positive for either	1.00		1.00	
Negative for both	1.16 (0.48–2.78)	0.74	2.81 (0.81–9.72)	0.10
HER2 status				
Positive	1.00		1.00	
Negative	0.97 (0.43–2.15)	0.93	0.68 (0.20–2.36)	0.55
Tumor stage				
T1	1.00		1.00	
T2	0.99 (0.28–3.56)	0.99	1.30 (0.15–11.22)	0.81
T3	1.44 (0.29–7.19)	0.66	1.18 (0.07–18.96)	0.91
T4	2.32 (0.61–8.89)	0.22	1.64 (0.18–14.97)	0.66
Nodal stage				
N0 or N1	1.00		1.00	
N2 or N3	2.64 (1.14–6.10)	0.02	3.40 (0.90–12.89)	0.07
Extent of disease				
<3 sites	1.00		1.00	
≥3 sites	1.32 (0.52–3.34)	0.55	3.45 (0.44–27.24)	0.24
Sites of metastasis				
Visceral	1.91 (0.79–4.61)	0.15	5.56 (0.70–44.09)	0.11
Non-visceral only	1.00		1.00	
Type of therapy				
Hormone therapy, immunotherapy, or both	1.00		1.00	
Chemotherapy alone or combined with other therapy	0.51 (0.15–1.77)	0.29	NE †	0.56
Maximum standardized uptake value				
Primary tumors	1.08 (1.00–1.15)	0.04	1.07 (0.98–1.17)	0.14
Regional lymph nodes	1.10 (1.02–1.20)	0.02	1.22 (1.07–1.40)	0.004
Total lesion glycolysis				
Primary tumors	1.00 (1.00–1.00)	0.57	1.00 (1.00–1.00)	0.80
Regional lymph nodes	1.01 (1.00–1.02)	0.04	1.02 (1.01–1.03)	0.002

CI, confidence interval; ECOG, Eastern Cooperative Oncology Group; HER2, human epidermal growth factor receptor type 2; NE, not evaluable; * Age was modeled as a continuous variable. † Because there was no occurrence of overall mortality in the ‘Hormone therapy, immunotherapy, or both’ group, coefficients did not converge and no models could be fitted.

## Data Availability

The data analyzed in the current study are available from the corresponding author on reasonable request.
